# Arterial oxygen saturation in healthy newborns delivered at term in Cerro de Pasco (4340 m) and Lima (150 m)

**DOI:** 10.1186/1477-7827-3-46

**Published:** 2005-09-12

**Authors:** Gustavo F Gonzales, Amelia Salirrosas

**Affiliations:** 1 Department of Biological and Physiological Sciences. Faculty of Sciences and Philosophy, Universidad Peruana Cayetano Heredia, Av. Honorio Delgado 430. Urb. Ingenieria. Lima, Peru. PO Box 1843. Lima, Peru; 2 Instituto de Investigaciones de la Altura. Universidad Peruana Cayetano Heredia, Lima, Peru

## Abstract

**Background:**

High altitude is associated with both low pulse oxygen saturation at birth and more pre-term deliveries. The present study was performed to determine pulse oxygen saturation in newborns at term in Cerro de Pasco (4340 m) and Lima (150 m) to test the hypothesis that low pulse oxygen saturation at birth at high altitudes was not observed at term deliveries.

**Methods:**

The present study was designed to determine pulse oxygen saturation values through 1 minute to 24 hours and values of Apgar score at 1 and 5 minutes in newborns delivered at term in Cerro de Pasco (4340 m) and Lima (150 m). Pulse oxygen saturation was recorded in 39 newborns from Cerro de Pasco (4340 m) and 131 from Lima (150 m) at 1, 2, 3, 4, 5, 10, 15, 30 minutes and 1, 2, 8 and 24 hours after delivery. Apgar score was assessed at 1 and 5 minutes after birth. Neurological score was assessed at 24 h of birth by Dubowitz exam.

**Results:**

Pulse oxygen saturation increased significantly from 1 to 15 min after birth at sea level and from 1 to 30 minutes at Cerro de Pasco. Thereafter, it increased slightly such that at 30 min at sea level and at 60 minutes in Cerro de Pasco it reached a plateau up to 24 hours after birth. At all times, pulse oxygen saturation was significantly higher at sea level than at high altitude (P < 0.01). At 1 minute of life, pulse oxygen saturation was 15% lower at high altitude than at sea level. Apgar score at 1 minute was significantly lower at high altitude (P < 0.05). Neurological score at 24 hours was also lower at high altitude than at sea level. Head circumference, and Apgar score at 5 minutes were similar at sea level and at high altitude (P:NS). Incidence of low birth-weight (<2500 g) at high altitude (5.4%) was similar to that observed at sea level (2.29%) (P:NS). Incidences of low pulse oxygen saturation (<30%), low Apgar score at first minute (<7) and low neurological score at 24 h (<19) were significantly higher at high altitude than at sea level (P < 0.0001; P < 0.0001; and P < 0.001, respectively).

**Conclusion:**

From these analyses may be concluded that pulse oxygen saturation at 4340 m was significantly low despite the fact that births occurred at term. Apgar scores at first minute and neurological scores were also lower at high altitudes.

## Background

Post-ductal pulse oxygen saturation (SpO_2_) in healthy newborns infants two minutes after birth was 67% in average and it takes 14 minutes to reach an oxygen saturation of 95% [1]. Harris et al [2] found at sea level a gradual rise in mean SpO_2 _value from 61% to 5 min after an uncomplicated vaginal birth.

Studies on pulse oxygen measurements during the first 30 minutes of life at high altitudes are few. Gonzales and Salirrosas [3] showed that mean pulse oxygen saturation at first minute of life was 41.3% in Cerro de Pasco, Peru (4340 m) compared with 60.4% in Lima (150 m). Ramirez-Cardich et al [4] (2004) measured pulse oxygen saturation at 10 minutes of life in La Oroya, Peru (3,800 m), but data were not compared with those obtained at sea level. All of the other studies are related to values after 30 minutes of life. In these reports, pulse oxygen saturation was significantly lower in infant populations at high altitudes around the world [5-10] as compared with values at sea level. Pulse oxygen saturation values in infants decreased as altitude increased. At 2640 m, In Bogota, Colombia, SpO_2 _in children aged 5 days to 24 months was 93.3%, whereas in El Alto, Bolivia (4018 m) the mean value of SpO_2 _in children aged 0–5 months was 87.3% [7]. Low arterial oxygen saturation at 30 min of life has also been observed in Morococha, Peru (4540 m) [11].

The importance to study pulse oxygen saturation in newborns at high altitude is that increased neonatal and infant mortality have been associated with reduced oxygen availability at high altitude [9]. SpO_2 _cutoff value of 30% has been considered reasonable in clinical trials as values below 30% were related to low umbilical arterial blood pH [12,13]. In Cerro de Pasco (4340 m), 20.3% of newborns had low pulse oxygen saturation (<30% SpO_2_) whereas in Lima the incidence was of 2.1% [3].

Why at the same oxygen partial pressure some individuals are more hypoxemic than others seems to be related to antiquity in the place of birth [5,9,14], high incidence of prematurity at high altitude [15] or intrauterine growth restriction [16].

Antiquity of the generations living at high altitudes seems to be related to the pulse oxygen saturation values [5,9,14]. To more antiquity the life at high altitude, the pulse oxygen saturation value will be higher. For instance, In Lhasa, Tibetan newborns had higher arterial oxygen saturation at birth and during the first four months of life than Han newborns. Native Tibetans have inhabited the Himalayan Plateau for approximately 25,000 years, whereas people of Han ancestry had moved there only 50 years ago [9].

In both Huancayo (3280 m) and Cerro de Pasco (4340 m) located in the Central Andes of Peru pre-term deliveries (<37 weeks of gestational age) were higher than at Lima (150 m) [15,17]. These findings contrast with large population-based studies which refer that altitude produce fetal growth restriction [16].

In Peru, populations from Central Andes as Cerro de Pasco are more recently inhabited (about 400 years) whereas those from the Southern Andes live there for more than 10,000 years [18]. The low antiquity of life at Cerro de Pasco or the high incidence of pre-term deliveries may explain the low pulse oxygen saturation values in newborn in that place.

In an attempt to control for the effect of pre-term deliveries, we have examined if pulse oxygen saturation in newborns at term in Cerro de Pasco (4340 m) at the Central Andes of Peru was different to that in another population at term born at sea level.

## Methods

The study was performed in 39 newborn infants immediately after birth in Cerro de Pasco and 131 in Lima. Sample size was calculated with a limit of confidence of 95% (z = 1.96) and a power of 15%. The main outcome variable to determine sample size was the incidence of low pulse oxygen saturation at Cerro de Pasco (20%) and low pulse oxygen saturation at Lima (2%) [3]. Minimum sample size was 35. With 10% of missing data, the minimum final sample size was 39 at each place. In the present study we have included 3 cases at sea level by each case at high altitude. For the analysis two infants from Cerro de Pasco were excluded because data from gestational age measured by Usher method was 37 and 38 weeks.

Deliveries in Lima (150 m) occurred at the Hospital Nacional María Auxiliadora and in Cerro de Pasco (4340 m) at the Hospital Daniel Alcides Carrion. Both are public hospitals attending by a population of low socioeconomic status. Mothers in the present study live in districts near the public hospitals, and these districts are classified as belonging to the low socioeconomic strata.

During the last 60 years, significant migration has occurred from the Peruvian highland to the coast [19]. Most of the migrants are settled in places named cones. One of these cones corresponds to the district in which the Hospital Maria Auxiliadora is located. From this, it results that population at sea level studied has the same ethnicity as those from Cerro de Pasco. In the present study 34.35% of mothers at sea level had at least one Quechua surname, whereas 27.94% of mothers at Cerro de Pasco had at least one Quechua surname.

Weeks of gestation for the mothers were 37–42 weeks (39.33 ± 0.12 weeks for Lima and 40.49 ± 0.16 weeks for Cerro de Pasco, mean ± SEM) based on the last normal menstrual period (LNMP). This value was confirmed with Usher method [20]. Mean vales for gestational age are observed in Table 1. It has been demonstrated that the large majority of deliveries occurring at or near term showed LNMP-based gestational age estimates that were valid within plus or minus seven days of the ultrasound estimates (Gold standard). There were excluded from the study neonates with malformations, and those whose parents did not accept voluntarily to participate in the study.

**Table 1:** Characteristics of the mothers at sea level and at high altitude (4340 m).

	Lima (n = 131)	Cerro de Pasco (n = 37)
	
	mean ± SEM	mean ± SEM
Age (years)	25.15 ± 0.58	25.46 ± 0.89
Body Weight (Kg)	54.98 ± 0.69	53.31 ± 1.13
Height (m)	1.55 ± 0.01	1.53 ± 0.01
Age at menarche (years)	13.16 ± 0.16	14.22 ± 0.25*
Hemoglobin at second trimester gr%	10.53 ± 0.27 (32)	13.42 ± 0.64 (6)*
Hemoglobin at third trimester gr%	10.87 ± 0.14 (80)	13.36 ± 0.42 (14)*
Gestational age (Usher) weeks	40.17 ± 0.03	39.51 ± 0.16*

Number of subjects is between parentheses. *P < 0.01 with respect to values in Lima (150 m)

Pediatricians, medical students or nurses provided post-birth care, and assigned an Apgar score at 1 and 5 minutes. No fetal monitoring and respiratory rate data were available.

Data on maternal age, body weight, height and age at menarche were obtained from the clinical records. In the cases where mother received pre-natal care, hemoglobin measurements were also recorded.

This study was approved by the Institutional Review Board of the Scientific Research Direction at the Universidad Peruana Cayetano Heredia.

### Techniques

Oximetry was performed using a pulse oximeter NELLCOR N-20 (Nellcor, Inc., Hayward, Calif) with a sensor cable model OC-3 for OXICLIQ-N sensor (oxygen transducer) applied to the first finger of the left foot immediately after delivery and after a segment of the umbilical cord was clamped. Post-ductal pulse oxygen saturation measurements were performed at 1, 2, 3, 4, 5, 10, 15, 30, 60 minutes, and 2, 8 and 24 hours after delivery. The sensor was changed almost every 8 neonates.

Oxygen saturation was monitored continuously by one of the researchers (AS) in both, Lima and Cerro de Pasco. The value observed at each time of monitoring was recorded. To assurance that readings were valid a regression analysis between pulse oxygen saturation at first minute of life and Apgar score at first minute was performed after controlling maternal age, gestational age, birth-weight, and height at birth.

The neurological evaluation was assessed at 24 hours after birth by using the neurological part of the Dubowitz's exam [21]. The neurological part of the exam was scored from 0 to 35. The neurological exam was applied when the newborn infant was in peaceful conditions. The Dubowitz exam included the following neurological criteria: posture, square window, ankle dorsiflexion, arm recoil, leg recoil, popliteal angle, scarf sign, heel to ear, head lag and ventral suspension.

The modified Dubowitz score was validated with the Scanlon neurobehavioral testing, which, it is specific for neurological assessment shortly after birth. The Scanlon test could be assessed as early as eight hours after birth [22]. The modified Dubowitz score (MDS) showed a significant correlation with Scanlon Neurobehavioral Testing: r = 0.64; P < 0.001 [23]. The test was performed by one of the researchers: AS.

### Statistical Analysis

Values for pulse oxygen saturation were averaged for each neonate according to place of study and time after birth. Data are reported as group means ± SEM. Data were analyzed by two-way ANOVA. Differences between pair of means were performed with the Scheffee test.

Low birth-weight was defined as values below 2500 g; low Apgar at first or fifth minute of life was defined as values below 7; low pulse oxygen saturation at first minute was defined as values <30%; low neurological scores at 24 h was defined when values were <19. Cases of low pulse oxygen saturation at first minute, low Apgar at first minute, and low neurological score were assessed as proportions. Differences between proportions were analyzed by Fisher's exact test. A multiple regression analysis was also performed to determine the independent effect of changes in SpO_2 _during first three minutes on neurological score.

A P-value was considered significant when was below 0.05.

## Results

### Characteristics of the mothers

Mothers that were included in this study had ages that ranged from 15 to 41 years (25.15 ± 0.58, mean ± SEM) in Lima and 16–42 years (25.46 ± 0.89) in Cerro de Pasco. Mother's ages, heights and body weights were similar at sea level and at high altitude (Table 1). Age of menarche was delayed at high altitude related to sea level (P < 0.01). Gestational age estimate by Usher method was 0.66 weeks lower at high altitude than at sea level (P < 0.01) (Table 1). Premature rupture of membranes was recorded in 20.6% of mothers at sea level and 16.2% at high altitude. At sea level, 87.03% of women had prenatal care, whereas at high altitude 70.27% of women received prenatal care.

Hemoglobin values were obtained at the second trimester in 32 cases in Lima, and 6 cases in Cerro de Pasco, and for the third trimester in 80 cases in Lima and 14 cases in Cerro de Pasco. Hemoglobin values at second and third trimester of pregnancy were also higher at high altitude than at sea level (P < 0.01).

### Characteristics of the newborns

Despite that both groups were conformed by newborns delivered at term (40–41 weeks of gestation), the birth-weight (P < 0.01), height (P < 0.01), and thoracic circumference (P < 0.05) were significantly lower at Cerro de Pasco (4340 m) than at Lima (150 m). Birth weight at high altitude represented in average 400 grams less than at sea level, whereas height represented 1.5 cm less at high altitude than at sea level. Apgar at first minute of life (P < 0.05), pulse oxygen saturation at first minute (P < 0.01), and neurological score at 24 hours (P < 0.01) were also lower at high altitude than at sea level. Head circumference, and Apgar score at 5 minutes were similar at sea level and at high altitude (P: NS) (Table 2). No infant included in the present study required oxygen therapy as part of delivery room care.

**Table 2:** Characteristics of the newborns delivered at 40–41 weeks of gestational age in Lima (150 m) and Cerro de Pasco (4340 m)

	Lima (n = 131)	Cerro de Pasco (n = 37)
	mean ± SEM	mean ± SEM
Birthweight (g)	3329.31 ± 34.84	2925.41 ± 43.63*
Height (cm)	50.54 ± 0.15	48.99 ± 0.22*
Head circumference (cm)	34.30 ± 0.11	34.31 ± 0.20
Thoracic circumference (cm)	33.55 ± 0.16	32.77 ± 0.22**
Apgar At first minute	7.95 ± 0.06	7.03 ± 0.35**
Apgar at five minutes	8.96 ± 0.03	8.68 ± 0.14
SpO_2 _at first minute (%)	60.60 ± 1.20	42.32 ± 2.54*
Neurological score at 24 h	27.44 ± 0.30	22.00 ± 0.67*

*P < 0.01, **P < 0.05 with respect to values in Lima (150 m)

Pulse oxygen saturation at first minute was significantly related to Apgar score at first minute of life after controlling for maternal age, gestational age, birth-weight and height at birth. The coefficient of determination was R^2 ^= 0.30, P < 0.003. At high altitude, SpO_2 _at first minute in neonates to mothers with prenatal care was similar to that obtained in neonates to mothers without prenatal care (41.13 ± 3.19%, mean ± SEM and 44.82 ± 3.75%, respectively; P:NS). Similarly, in cases of premature rupture of membranes, the SpO_2 _in neonates at first minute of life was 43.25 ± 2.88% similar to that obtained when premature rupture of membranes was present, 40.10 ± 4.86% (P:NS).

Incidence of neonates with low birth-weight (<2500 g) at high altitude (5.4%) was similar to that observed at sea level (2.29%) (P:NS). Incidence of low pulse oxygen saturation (<30%), low Apgar score at first minute (<7) and low neurological score at 24 h (<19) was significantly higher at high altitude than at sea level (Odds Ratio, OR: 8.76, 9.96, and 35.86, respectively) (Table 3).

**Table 3:** Incidence of low birth-weight, low pulse oxygen saturation at first minute, low Apgar at first minute, and low neurological score at 24 hours.

Incidence of	Lima	Cerro de Pasco	OR	Confidence Interval (at 95%)	P
Low birthweight	3/131 (2.29%)	2/37 (5.40%)	2.44	0.39–15.18	0.3
Low pulse oxygen saturation	4/131 (3.05%)	8/37 (21.62)	8.76	2.47–31.07	0.0001
Low Apgar at first minute	3/131 (2.29%)	7/37 (18.92%)	9.96	2.43–40.77	0.0001
Low neurological score at 24 h	1/131 (0.76%)	8/37 (21.62%)	30.33	3.60–255.92	0.0001

Cases are in the numerator. OR: Odds Ratio. P: Probability.

### Pulse oxygen saturation in newborn infants

**Figure 1:**
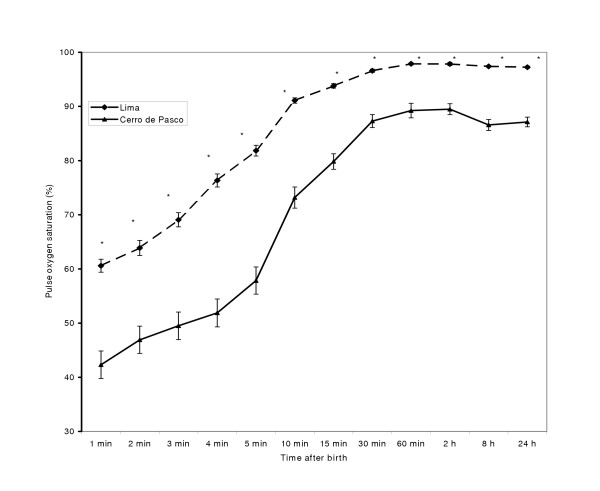
Mean pulse oxygen saturation in newborns at 40–41 weeks of gestational age at sea level and at high altitude (4340 m). Data are mean ± SEM. F value = 2146.97, P < 0.0001 (Two-way ANOVA test).*P < 0.001 with respect to values at sea level.

Figure 1 shows SpO_2 _measured by pulse oximetry from 1 minute to 24 hours of life. Oxygen saturation increased with high slope from 60.60 ± 1.20% (mean ± SEM) at first minute of life to 91.10 ± 0.5% at 15 minutes of life in Lima and from 45.08 ± 2.47% at first minute to 87.56 ± 1.19% at 30 minutes of life in Cerro de Pasco. Thereafter a plateau was observed. At all times SpO_2 _values were higher at sea level than at high altitude (P < 0.01). According to the regression analysis, SpO_2 _increases with time after birth (4.11 ± 0.07, coefficient of regression ± standard error; P < 0.0001) and decreases with altitude (-63.90 ± 0.98; P < 0.0001). The coefficient of determination for the model was 0.64. When the interaction place of birth X time after birth is included in the model, the increase in the coefficient of determination (R^2 ^= 0.65) is negligible. However, this interaction was significant (1.19 ± 0.14; coefficient of regression ± standard error; P:0.0001).

### Probability for low neurological score at 24 hours of life at high altitude

Almost all of the components of the Dubowitz score were significantly lower at high altitude than at sea level. At high altitude score of the leg recoil test was not related to the Dubowitz score (r = 0.28; P:NS). The highest correlation was observed with scarf sign test (r = 0.68, P < 0.001), popliteal angle (r = 0.63, P < 0.001), posture (r = 0.60, P < 0.001).

At high altitude, neurological score at 24 hours was related to SpO_2 _values at first minute of life (8.09 × 10^-2 ^± 0.04; coefficient of regression ± SE, P < 0.05). In Table 4 is presented data from the multivariate analysis to assess the independent effect of change of SpO_2 _from 1st to 3^rd ^minute of life on neurological score at 24 hours. Data showed that low neurological score was related to dilatation period (P < 0.05) but not to changes in SpO_2 _from first to third minute of life (P:NS).

**Table 4:** Multiple regression analysis to assess the independent effect of pulse oxygen saturation during firsts minutes of life on neurological score at 24 hours of birth in Cerro de Pasco (4340 m).

**Neurological score**	**β ± SE**	**P**	**Confidence Interval at 95%**	
SpO_2 _3 min - SpO_2 _1 min	3.18 × 10^-2 ^± 0.03	NS	-0.029	0.093
Period of dilatation	0.24 ± 0.11	<0.05	0.017	0.463
Constant	16.22 ± 2.62	<0.001	10.90	21.539

β ± SE: Coefficient of Regression ± standard error. P = Level of significance.

## Discussion

Monitoring of pulse oxygen saturation is useful for the care of infants and children [24]. We had studied pulse oxygen saturation in a selected population of newborns delivered at term in Lima (150 m) and Cerro de Pasco (4340 m) in the Central Andes of Peru. Results demonstrated that pulse oxygen saturation was significantly lower at high altitudes than at sea level since first minute of life.

A rapid increase in SaO_2 _must occur within the first few minutes of extra-uterine life if viability is to be maintained. In the present study we have demonstrated that pulse oxygen saturation increases significantly from 1 minute to 15 minutes at sea level and from 1 to 30 minutes at high altitude. Others obtained similar findings in populations at sea level [1,2].

It is possible that different factors may affect pulse oxygen readings as meconium-stained amniotic fluid, child's activity during the SpO_2 _measurements, and an anemic condition of the mothers. In a recent study using the Nellcor pulse-oximeter, in cases with meconium as compared to clear amniotic fluid, the pulse oximetry remained unchanged [13]. The effect of sleep or awake during measurement in children between 24 hours of life was negligible. Only after one week of age do activities influence SpO_2 _[9]. With respect to anemic condition, our small sample may not allow an adequate analysis on anemic and non-anemic women. However, another study had demonstrated that SpO_2 _levels and respiratory rates of infant natives at 3750 meters were not different between infants born to non-anemic and anemic mothers [4]. We also do not found impact of pre natal care or premature rupture of membranes on SpO_2 _values of neonates at first minute of life.

Low oxygenation may have some implications on neurological development, particularly in cases when low oxygenation was prolonged. In fact 0.76% of newborns at sea level showed low neurological score at 24 hours, compared to 21.62% in Cerro de Pasco at 4340 m. These incidence values are similar to those observed for the incidence of low pulse oxygen saturation at minute 1 of life.

It is not clear whether low oxygen saturation at high altitude occurred intra-partum or whether it was an accumulative effect from intrauterine life. It has been observed that a single prolonged episode of fetal hypoxia may produce neurological abnormality [25]. We have previously demonstrated that at sea level maintaining low oxygen saturation for at least three minutes the neurological score at 24 hours of life was low [23]. However, the situation in Cerro de Pasco was different. In fact, changes of SpO_2 _from 1 to 3 minutes were not related to neurological score at 24 hours. Instead, SpO_2 _at first minute or dilatation period were related to the neurological score at 24 hours.

It is interesting the finding that incidence of low birth-weight (2500 g) in these selected populations at sea level and at high altitude was similar. However, the sample is small to make a statement. In fact, mean birth weight was significantly lower at high altitude than at sea level (400 g less in average). This suggests that a restriction in growth occurs at high altitude. This has been described by other authors [26-28]. Low mean birth-weight at high altitude of Cerro de Pasco in newborns at term seems to be related more to the low-pressure exposure than for socio-economic differences [29,30]. It has been suggested that altitude acts as an independent factor in determining birth weight with a reduction of 100 gr. per 1000 m elevation gain [31]. In our study, a difference of 400 grams less in average was observed in birth weight at 4340 m than at sea level.

Our data also demonstrated that head circumference was similar at sea level and at high altitude despite that low average birth-weight and height. Several studies have demonstrated that head size is relatively well preserved whereas body weight is significantly reduced at high altitudes [30,32].

The Apgar score is the traditional and most universal criterion for assessing the newborn's well being in the delivery room. The data of this study indicate that Apgar score at first but not at fifth minute was lower at high altitude than at sea level. This was also associated with a significantly high incidence of low Apgar score at first minute in high altitude (18.92%) than at sea level (2.29%) neonates. This finding is confirmatory with our previous results of higher incidence of low Apgar at Cerro de Pasco [15].

We have previously demonstrated that Apgar score at 1 minute but not at 5 minutes was related to neurological evaluation at 24 hours [23]. This means that infants with low Apgar score at first minute may recover at 5 minute but it does not improve neurological evaluation at 24 h of life. Low SpO2 (<30%) has been associated with acidemia [12]. Low arterial blood pH (<7.01) at birth was significantly related to low Apgar score [33]. Then it is not surprising that SpO_2 _at first minute was related to Apgar score at first minute and with low neurological score at 24 hours of life.

In summary, the study demonstrates that after controlling gestational age in the design pulse oxygen saturation and Apgar scores at 1 minute in newborns delivered at term were lower at high altitude in the Central Andes than at sea level.

## Authors' contributions

GFG conceived of the study, and participated in its design and coordination and drafted the manuscript.

AS participated in the design of the study, the work of field and the statistical analysis.

All authors read and approved the final manuscript.
